# Audiological findings in aphasic patients after stroke

**DOI:** 10.1590/S1679-45082014AO3119

**Published:** 2014

**Authors:** Solange Satie Onoue, Karin Zazo Ortiz, Thaís Soares Cianciarullo Minett, Alda Christina Lopes de Carvalho Borges

**Affiliations:** 1 Universidade Federal de São Paulo, São Paulo, SP, Brazil.

**Keywords:** Aphasia/etiology, Stroke/complications, Hearing loss/etiology, Hearing loss, sensorineural/etiology

## Abstract

**Objective:**

To outline the audiological findings of aphasic patients after cerebrovascular accidents.

**Methods:**

This is a cross-sectional study performed between March 2011 and August 2012 in the Speech, Language, and Hearing Pathology Department of the *Universidade Federal de São Paulo*. A total of 43 aphasic subjects (27 men) were referred for audiological evaluation after stroke, with mean age of 54.48 years. Basic audiological evaluation tests were performed, including pure tone audiometry, speech audiometry (speech recognition threshold and word recognition score), immittance measures (tympanometry and contralateral acoustic reflex), and transient otoacoustic emissions.

**Results:**

Sensorineural hearing loss was prevalent (78.6%). Speech recognition threshold and word recognition score were not obtained in some patients because they were unable to perform the task. Hearing loss was a common finding in this population.

**Conclusion:**

Comprehension and/or oral emission disruptions in aphasic patients after stroke compromised conventional speech audiometry, resulting in the need for changes in the evaluation procedures for these patients.

## INTRODUCTION

Cerebrovascular accidents (CVA) refer to a group of vascular disorders that affect the brain and compromise neurological function. CVAs are one of the top three most common causes of death in a majority of developed and developing nations.^(1)^ In Brazil, CVAs are the main cause of death.^(2,3)^

The risk of both hearing loss and CVAs increases with age.^(4)^ Furthermore, when a patient presents with a CVA, hearing loss can impede and affect both the evaluation process and speech rehabilitation, as several linguistic skills depend on peripheral and central auditory perception. We know that comprehension is always impacted in aphasic post-CVA patients to varying degrees^(5)^ because it is a widely reported central component,^(6)^ but it is not always clear whether some component of peripheral hearing is also damaged or how this component could affect the central processing of acoustic information. However, systemic studies on how to measure peripheral auditory acuity in aphasic individuals are lacking.

## OBJECTIVE

To investigate the occurrence of peripheral hearing loss in aphasic post-CVA patients.

## METHODS

This was a cross-sectional study performed between March 2011 and August 2012 in the Speech, Language, and Hearing Pathology Department of the *Universidade* *Federal de São Paulo* (UNIFESP), and approved by the Research Ethics Committee (protocol 1272/02). After receiving full information about the study, written informed consent was obtained from all enrolled subjects.

All of the patients who were evaluated in the acquired speech and language disorder clinic at UNIFESP and diagnosed with aphasia after a single ischemic cerebral lesion in the left hemisphere, confirmed by neuroimaging, were selected for this study. Aphasic patients who agreed to audiology examination underwent otoscopy. After otoscopy, a medical history was taken, followed by the tests that make up the basic audiologic evaluation. Individuals who referred hearing deficits prior to stroke, noise exposure, or use of drugs that could interfere in audiological results were excluded from the study. Then the patients were exposed to pure tone audiometry, speech audiometry: speech recognition threshold (SRT) and the word recognition score (WRS), acoustic immittance (tympanometry and contralateral acoustic reflex threshold), and transient evoked otoacoustic emissions (TEOAE).

Auditory sensitivity was tested for the following frequencies: 250, 500, 1,000, 2,000, 3,000, 4,000, 6,000, and 8,000Hz, using a Midimate 622 audiometer coupled to a Sony CD player. An audibility threshold of 25dB was considered normal hearing for all frequencies.^(7)^ For the SRT test, which identifies the threshold for detecting speech, a CD recording containing a list of disyllabic words was used.^(8)^ SRT was considered compatible when it was equal to or up to 10dB above the average of auditory thresholds at 500, 1,000 e 2,000Hz. WRS was analyzed for compatibility in relation to average auditory thresholds at frequencies of 500, 1,000, and 2,000Hz according to the following criteria: for an average between zero and 10dB, an SRT of 100% was considered compatible; between 11 and 25dB, 96% accurate or greater; between 26 and 40dB, 82% or greater; between 41 and 55dB, 66% or greater; between 56 and 70dB, 56% or greater; between 71 and 90dB, 26% or greater, and for an average over 90dB, 8% accurate or greater.

The WRS, which measures the patient’s auditory acuity, was assessed by providing speech stimuli organized into four lists^(9)^ and recorded on a CD. For aphasic patients who exhibited modified expression of verbal symbols, we used a picture album and instructed the patients to point to the word they heard.

Next, the patient’s tympanometry and the acoustic reflex threshold of their stapedius on the contralateral side were measured at 500, 1,000, 2,000, and 4,000Hz. Their acoustic reflex was considered normal when the difference between their audibility limit and reflex limit was between 70 and 90dBNA for at least three frequencies.

The OAE was considered normal when OAE results were positive and the signal-to-noise ratio and the overall response were greater than or equal to 3dB. The minimum value for the stability and reproducibility parameters were 70 and 50%, respectively.^(10)^ The stimulus used was adjusted at the ear canal and delivered at 80dB.

### Data analysis

The χ^2^ (without Yates correction) was used for categorical comparisons of the data. Differences in the means of continuous measurements were tested by Student’s *t*-test, followed by the Mann-Whitney test, which without exception did not identify any discrepant results (only the parametric test results will be reported). All statistical analyses were performed on a personal computer using the Statistical Package for the Social Sciences (SPSS) version 11.5.1 for Windows.

## RESULTS

Fifty patients in the language acquired disorders outpatient clinic who were diagnosed with strokes and who presented aphasia were selected. A total of seven were excluded (two were unable to complete the protocol, and five were absent). The remaining 43 patients met the inclusion criteria. Therefore, 86 ears were examined.

### General characteristics

Sixteen of the 43 patients were women, and the group had a mean age of 57.6 (standard deviation − SD=13.2) years.

### Thresholds

There were no statistically significant differences between the threshold means of the right and left ears for any of the frequencies tested (data not shown).

The audiogram was not performed in five ears: two individuals could not understand the test instructions, and in one ear, the external acoustic meatus was obstructed.

Table 1 presents the audibility thresholds for the frequencies of 250, 500, 1,000, 2,000, 3,000, 4,000, 6,000, and 8,000Hz.

**Table 1:** Average audibility thresholds for both ears

	250Hz	500Hz	1KHz	2KHz	3KHz	4KHz	6KHz	8KHz
Mean	22.8	20.8	17.3	20.8	24.3	33.5	36.9	33.4
SD	11.0	10.8	11.8	16.8	17.2	21.4	22.1	25.5
Median	20	20	15	20	20	30	35	30
Minimum	5	5	5	0	0	5	0	0
Maximum	65	60	65	85	80	100	100	95
n=81								

SD: standard deviation.

The audiogram was abnormal in 56 ears, indicating some type of hearing loss. Of the 81 ears, 25 had normal tonal thresholds at all frequencies, and 56 ears had hearing loss for at least one frequency.

The following types of hearing loss were observed: 78.6% exhibited sensorineural loss, 17.8% exhibited hearing loss at just one frequency, and 3.6% exhibited mixed loss. Of the audiograms for patients with sensorineural hearing loss, we observed a descending curve in 50% of cases, a flat curve in 19.6%, an ascending curve in 1.8%, and other types in 28.6%.

### Speech recognition thresholds

The average values for the SRT were 28.6 (SD=12.6) for the right ear and 23.1 (SD=7.85) for the left ear. Pictures had to be used to obtain the SRT in 12.8% of the sample (11 ears).

### Logoaudiometry

The WRS was consistent with the pure-tone threshold audiometry results in 42.4% of the tested ears, and 15.3% of the patients used pictures. However, the WRS was inconsistent in 57.6% of the cases because the possibility to respond to speech was much worse, considering the auditory thresholds obtained with these patients in relation to average auditory thresholds at frequencies of 500, 1,000, and 2,000Hz. The previously established criteria were: for an average between zero and 10dB, an SRT of 100% would be considered compatible; between 11 and 25dB, 96% accurate or greater; between 26 and 40dB, 82% or greater; between 41 and 55dB, 66% or greater; between 56 and 70dB, 56% or greater; between 71 and 90dB, 26% or greater; and for an average over 90dB, 8% accurate or greater.

Logoaudiometry was performed using the conventional method in 63 ears (73.3%) and with pictures in 11 ears (12.8%). There was no response after repeating the words or pointing to pictures in 12 of the tested ears (14%).

### Acoustic immittance (tympanometry)

Acoustic immittance measurements generated type A tympanometric curves in 97.6% of the ears. Type C curves were noted in only two ears (2.4%). Type B curves were not observed.

### Acoustic reflex

Acoustic reflex data were analyzed by measuring the difference between the auditory limit and the acoustic reflex limit at the tested frequency.

Table 2 presents the acoustic reflex results for both ears.

**Table 2:** Averages for the difference between auditory and acoustic reflex thresholds in both ears

Acoustic reflex	500Hz	1kHz	2kHz	4kHz
Mean	73.68	75.83	74.20	67.92
Median	75	75	75	70
Standard deviation	10.58	10.79	12.91	18.05
Minimum	50	55	35	30
Maximum	100	105	105	95
Sample size	72	70	69	65
Lower limit	71.24	73.30	71.16	63.54
Upper limit	76.12	78.36	77.25	72.31

Next, we classified the difference between the reflex and audibility thresholds into four categories and compared each frequency. These data are presented on table 3.

**Table 3:** Distribution of the values between the reflex and audibility thresholds at each of the tested frequencies

Reflex	500Hz	1kHz	2kHz	4kHz
	n (%)	n (%)	n (%)	n (%)
Less than 70	21 (24.4)	17 (19.8)	17 (19.8)	31 (36.0)
From 70 to 90	49 (57.0)	49 (57.0)	49 (57.0)	32 (37.2)
More than 90	2 (2.3)	5 (5.8)	3 (3.5)	2 (2.3)
Absent	14 (16.3)	15 (17.4)	17 (19.8)	21 (24.4)

The OAE values did not differ between ears. The results in both ears for all individuals, including the confidence intervals, are shown on table 4.

**Table 4:** Average values for the amplitude of the transient evoked otoacoustic emissions response in both ears

TEOAE	1kHz	1.5kHz	2kHz	3kHz	4kHz
Mean	7.96	9.40	7.36	7.34	7.16
Median	6	9	6	7	7
Standard deviation	4.67	4.81	4.98	4.68	4.73
Minimum	1	1	1	1	1
Maximum	22	22	21	16	16
Sample size	45	55	45	29	19
Lower limit	6.59	8.13	5.90	5.64	5.03
Upper limit	9.32	10.67	8.81	9.05	9.28

TEOAE: transient evoked otoacoustic emissions.

## DISCUSSION

The most significant finding in this study was the high rate of hearing loss, most commonly sensorineural, observed in this population of aphasic patients. Tonal audiometry thresholds proved to be an extremely useful procedure, but the aphasic patients’ difficulties in understanding and/or vocalizing affected the conventional logoaudiometry test and emphasized the need for alternative evaluation methods for this population.

The sample population had an average age of 57.48 years, similar to reports of other studies,^(11,12)^ although some studies have reported CVAs in patients over 60 years of age.^(13,14)^

Two individuals were unable to complete the conventional tests because they did not understand the instructions. These aphasic patients exhibited severe comprehension problems.

In 94.1% of the cases, we were able to evaluate hearing using conventional tonal audiometry. Therefore, we can verify that conventional tonal audiometry is a valid method for evaluating this population and can be used to determine the degree of peripheral hearing loss that can aggravate the comprehension disorders common in patients with aphasia.

The usual tonal thresholds for high frequencies are higher than the average for low frequencies. The cochlea is a helicoid structure with approximately 2 and 2/3 turns. The base of the cochlea tends to vibrate at high frequencies, and the apex tends to vibrate at lower frequencies. Thus, lesions begin at high frequencies. In addition, presbycusis is caused by deficiencies in blood flow to the inner ear, causing degenerative lesions in the organ of Corti. The initial lesion causes deafness for acute sounds (descending curve) and loss of word discernment. The age of the population ranged from 22 to 82 years, and although the average age of the study population was 57.48 years, part of the sample may experience this type of hearing loss simply due to age.^(15)^ Presbycusis is represented by four subtypes: sensorial (loss of hair cells), neural (loss of spiral and ganglion neurons), metabolic (atrophy of the stria vascularis), and mechanical (thickening and stiffening of the basilar membrane).^(16)^ However, because multiple genetic and environmental factors cause presbycusis, most cases are of mixed pathology and affect multiple cell types.^(17) ^ This considered, we could hypothesize that patients with vascular problems could also be more susceptible to these mechanisms.

In addition, because CVA involves vascular changes, it is possible that these adults with vascular problems could exhibit early blood flow deficiencies in the inner ear.^(18)^ Therefore, vascular problems could lead to compression and subsequent damage to a specific vascularized area, and the individual may exhibit some symptoms arising from the lack of blood flow to this area.^(19)^

Audiologic evaluation results revealed a normal audiogram in 25 ears; 56 ears exhibited some type of hearing loss. In the identified hearing loss cases, 78.6% were sensorineural, and 17.8% had an isolated decrease at a specific frequency (250, 6,000, and/or 8,000Hz), indicating that middle ear changes are rare in these patients. Only two ears (3.6%) exhibited mixed-type hearing loss.

In the audiogram shape test, the most common result was a descending curve. This may be because part of the sample (47.72%) consisted of individuals older than 60 years of age, as mentioned previously, and these subjects may have exhibited presbycusis, which is normally characterized by sensorineural hearing loss with a descending curve.^(19)^

SRT measurements require the patients to repeat the words heard. We know that this population of post-CVA aphasic patients may exhibit changes in their vocalization and often exhibit specific changes in their ability to repeat.^(3,20)^ These changes may affect their responses in this type of test. Therefore, pictures had to be used in 12.8% of the tests. We know that a SRT measured using a picture album is potentially easier, likely because it is a closed set (multiple choice) with a limited number of alternative responses. Thus, post-CVA aphasic patients are expected to exhibit better results than those using an open set.^(21)^ However, even though it is a simpler procedure, an SRT with a picture album had to be used in this population of aphasic patients whose difficulties vocalizing could potentially hinder SRT data collection.

We were also unable to obtain an SRT in 14% of the tested ears because some patients did not understand the test’s instructions or the stimuli due to severe comprehension difficulties, which made it impossible to repeat or to point to a picture of the words heard.

A picture album was also necessary during the WRS test in 15.3% of cases (13 ears), more often than the SRT test (12.8%), possibly because monosyllabic words with and without meaning were used in the WRS, making the task more difficult. Moreover, as observed in the SRT test, 14% were unable to repeat or point to pictures of the words heard. In addition, individuals were able to repeat the words in 57.6% of the cases, but the results were below the expected results for the degree of hearing loss. Even in the 37.7% of cases in which the audiogram and WRS were consistent, 15.3% used the picture album to respond. In aphasic patients, comprehension and/or vocalization are highly variable. Thus, it is possible that the aphasic patient understands the task but is unable to repeat or paraphrase the word, which is considered an error in the WRS test. The patient may also not be able to understand the test instructions, or the patient may understand the instruction but fail during linguistic input processing (auditory phonological analysis, phonological input buffer, phonological input lexicon, and semantic system).^(22,23)^ Therefore, if we take the complexity of linguistic processing into account, the observed errors during repetition could have multiple sources in aphasic patients. Although the repetitions are extremely simple for some people with a cerebral lesion, repetition is extremely complex in post-CVA aphasic individuals. In summary, the task of repeating words could be altered at multiple points in linguistic processing and/or could be associated with difficulties in speech motor programming, as is often found in patients with aphasia.^(24,25)^ In patients with cerebral lesions and peripheral hearing impairment, the audiogram and WRS were consistent. The inconsistency between the audibility thresholds and the WRS in aphasic patients is related to the linguistic processing that generates errors in the task of repetition. These results have led us to rethink our methods for evaluating hearing in specific patients to remove the effect of the challenges experienced by these patients. Our results also confirm the importance of pointing tests, as patients with severe comprehension disorders respond better to this type of task.^(26)^

Type A tympanometric curves were the most commonly observed (97.6%), indicating that this population rarely has problems with the middle ear. The studied population includes individuals older than 60 years (47.72%), and sensorineural hearing loss accompanied by type A tympanometric curves is more common in this group, as they often arise from changes in the inner ear and/or central auditory pathways.^(19)^

The acoustic reflex analysis focused on the values of the difference between the acoustic reflex threshold and the audibility threshold at the tested frequencies. In this dataset, the average values for the highest frequency (4,000Hz) are lower than the average for the other frequencies. These results can be explained by the high number of people with sensorineural hearing loss with a descending curve in this sample. Recruitment is occasionally observed in the presence of hearing loss. Recruitment is a clinical symptom of cochlear hearing loss related to the rapid and abnormal psychoacoustic sensation of intensity that accompanies an increase in the intensity of the stimulus.

For all of the tested frequencies, the difference between the acoustic reflex threshold and the audibility threshold was between 70 and 90dB in most cases. At the frequencies of 500, 1,000, and 2,000Hz, there was a notable difference between this category and the others, but at 4,000Hz, we noticed a significant number of people were in the category below 70dB, indicating that recruitment was more common at 4,000Hz (a strong indicator of cochlear lesions), consistent with the fact that there was greater hearing loss at higher frequencies.

During the otoacoustic emission analysis, the average response at 4,000Hz was less frequent. In these tests, low responses were observed when the audibility limit for the frequency was near the limits for normality. In fact, the emission amplitude studied decreased as frequency increased.^(27)^ Because the majority of patients exhibited decreasing curves, the thresholds at high frequencies were higher than the thresholds at lower frequencies.

A comparison of the tonal audiometry and the TEOAE tests demonstrated that 19 patients lacked a TEOAE at one or more frequencies even with normal audibility thresholds. In fact, previous studies in individuals exposed to noise and individuals who underwent cisplatin chemotherapy treatment^(28-30) ^have demonstrated the importance of this evaluation technique for the early detection of changes in the cochlear cells, even when there has been no change in their audibility threshold levels measured by pure-tone threshold audiometry. Therefore, we suggest that this is a highly legitimate test, as it detects early cochlear changes that may occur as a result of blood flow loss. The test is also important for providing an idea of the patient’s hearing when they are unable to perform any of the activities in the basic audiologic evaluation.

In this study, we observed hearing loss at one or more frequencies in 56 ears, indicating a high prevalence of hearing loss in this population. We also observed that, of these 56 ears, 43 experienced sensorineural hearing loss, and 2 exhibited mixed-type hearing loss. The most common audiometric configuration was descendent. Individuals with high frequency hearing loss (higher than 2,000Hz) can exhibit difficulties recognizing speech.

Given the importance of hearing for developing linguistic skills and for the efficiency of communication, we conclude that audiologic evaluation in this population is indispensible, both for clinical and research reasons, as it is essential to differentiate peripheral and central deficits for the evaluation and rehabilitation of aphasic patients after a cerebral lesion.

### Limitations of this study

This is a cross-sectional study, so it is not possible to identify precisely the cause of hearing loss. In this study we were only able to show that the majority of aphasic patients presented with hearing loss.

## CONCLUSION

There was a high rate of hearing loss, most often sensorineural, in aphasic patients evaluated in this study. Changes in comprehension and/or vocalization in these patients affected their convention logo audiometry results and highlighted the need to use alternative evaluation procedures in this population in order to provide better assistance and better speech/language rehabilitation in this population.
