# Supplementary material for: Audiological findings in aphasic patients after stroke
Source: Einstein (Sao Paulo). 2014 Oct-Dec;12(4):433–9. doi: 10.1590/S1679-45082014AO3119 (PMC4879908; doi:10.1590/S1679-45082014AO3119)
Supplement: Translation

# Translation: Achados audiológicos em pacientes afásicos após acidente vascular encefálico

**Authors:** Solange Satie Onoue, Karin Zazo Ortiz, Thaís Soares Cianciarullo Minett, Alda Christina Lopes de Carvalho Borges

**Affiliations:** 1 Universidade Federal de São Paulo, São Paulo, SP, Brasil.

**Keywords:** Afasia/etiologia, Acidente vascular cerebral/complicações, Perda auditiva/etiologia, Perda auditiva neurossensorial/etiologia

## Abstract

**Objetivo:**

Avaliar os achados audiológicos em pacientes afásicos após acidente vascular encefálico.

**Métodos:**

Trata-se de um estudo transversal, realizado entre março de 2011 e agosto de 2012 no Departamento de Fonoaudiologia da Universidade Federal de São Paulo. Participaram do estudo 43 pacientes afásicos (27 homens) após acidente vascular encefálico, com média de idade de 54,48 anos. Foram realizados testes que compõem a bateria da avaliação audiológica básica: audiometria tonal liminar, logoaudiometria (limiar de reconhecimento de fala e índice percentual de reconhecimento de fala), medidas de imitância acústica (timpanometria e pesquisa do reflexo acústico contralateral) e emissões otoacústicas transitórias.

**Resultados:**

A perda auditiva neurossensorial foi prevalente (78,6%). Não foi possível obter o limiar de reconhecimento de fala e o índice percentual de reconhecimento de fala em todos os pacientes, pois alguns eram incapazes de realizar a tarefa. A perda auditiva foi um achado comum nessa população.

**Conclusão:**

As alterações de compreensão e/ou emissão oral apresentadas por estes pacientes afetaram a logoaudiometria convencional e apontam para a necessidade de uso de outros procedimentos de avaliação nessa população.

## INTRODUÇÃO

Acidentes vasculares cerebrais ou encefálicos (AVE) correspondem a um grupo de distúrbios vasculares que afetam o cérebro e comprometem a função neurológica. Os AVEs estão dentre as três causas de morte mais frequentes na maioria de países desenvolvidos e em desenvolvimento.^(1)^ No Brasil, os AVEs são a principal causa de morte.^(2,3)^

Com o avanço da idade, há um aumento do risco tanto para o AVE como para a deficiência auditiva.^(4)^ Além disso, quando o paciente tem um AVE, a perda auditiva pode impedir e afetar tanto o processo de avaliação quanto a reabilitação da fala, já que várias habilidades linguísticas dependem da percepção auditiva periférica e central. Sabemos que a compreensão está sempre afetada em pacientes afásicos após o AVE em graus variados,^(5)^ estando o componente central bem descrito,^(6)^ mas não está claro se algum componente da audição periférica também está alterado, ou de que forma tal componente poderia afetar o processamento central de informação acústica. Apesar disso, faltam estudos sistemáticos que revelem como se encontra a acuidade periférica em indivíduos com afasia.

## OBJETIVO

Investigar a ocorrência de perda auditiva periférica em pacientes afásicos após acidente vascular encefálico.

## MÉTODOS

Este foi um estudo transversal conduzido entre março de 2011 e agosto de 2012, no Departamento de Fonoaudiologia da Universidade Federal de São Paulo (UNIFESP), e aprovado pelo Comitê de Ética em Pesquisa (protocolo 1272/02). Após receber todas as informações sobre o estudo, todos os pacientes assinaram o Termo de Consentimento Livre e Esclarecido.

Todos os pacientes avaliados no ambulatório de distúrbios adquiridos de fala e de linguagem da UNIFESP e diagnosticados como tendo uma afasia após uma lesão isquêmica cerebral única no hemisfério esquerdo, confirmada por exame de neuroimagem, foram selecionados para este estudo. Os pacientes afásicos que compareceram para a avaliação audiológica foram submetidos à meatoscopia. Após a meatoscopia, foi feita a anamnese, seguida dos exames de compõem a avaliação audiológica básica. Os indivíduos que referiram deficits auditivos anteriores ao AVE, exposição a ruído, ou uso de drogas que poderiam interferir nos resultados audiológicos foram excluídos do estudo. Em seguida, os pacientes foram expostos à audiometria tonal liminar, logoaudiometria: limiar de reconhecimento da fala (LRF) e o índice percentual de reconhecimento de palavra (IPRF); medidas de imitância acústica (timpanometria e limiar do reflexo acústico contralateral); e emissões otoacústicas evocadas transitórias (EOAT).

A sensibilidade auditiva foi testada para as seguintes frequências: 250, 500, 1.000, 2.000, 3.000, 4.000, 6.000 e 8.000Hz, usando um audiômetro Midimate 622 acoplado a um CD *player *Sony. O limiar de audibilidade de 25dB foi considerado normal para todas as frequências.^(7)^ Para o LRF foi usada uma gravação em CD contendo um a lista de palavras dissilábicas.^(8)^ Esse teste foi considerado compatível quando era igual a ou até 10dB acima da média de limiares auditivos em 500, 1.000 e 2.000Hz. O índice de reconhecimento de palavra foi analisado quanto à compatibilidade em relação aos limiares auditivos médios, nas frequências de 500, 1.000 e 2.000Hz segundo os seguintes critérios: para uma média entre 0 e 10dB, foi considerado compatível um limiar de reconhecimento da fala de 100%; entre 11 e 25dB, 96% ou maior; entre 26 e 40dB, 82% ou maior; entre 41 e 55dB, 66% ou maior; entre 56 e 70dB, 56% ou maior; entre 71 e 90dB, 26% ou maior e, para uma média acima de 90dB, 8% ou maior.

O IPRF, que mostra a acuidade auditiva do paciente, foi obtido por meio da apresentação de estímulos de fala organizados em quatro listas^(9)^ gravadas em CD. Para pacientes afásicos que apresentavam alteração na emissão dos símbolos verbais, usamos um álbum de figuras e os instruímos a apontar para a figura da palavra ouvida.

Posteriormente, foi realizada a timpanometria e a pesquisa do limiar de reflexo acústico do estapédio, modo contralateral nas frequências sonoras de 500, 1.000, 2.000 e 4.000Hz. O reflexo acústico foi considerado normal quando a diferença entre o limiar de audibilidade e o limiar do reflexo estava entre 70 e 90dBNA em pelo menos três frequências.

O teste de EOAT foi considerado normal quando os resultados eram positivos, e a relação sinal-ruído e a resposta geral eram maiores ou iguais a 3dB. Os valores mínimos para os parâmetros de estabilidade e reprodutibilidade foram de 70 e 50%, respectivamente.^(10)^ Quanto ao estímulo, consideramos o nível mais próximo de 80dB.

## ANÁLISE DE DADOS

O teste χ^2^ (sem a correção de Yates) foi usado para comparações categóricas dos dados. As diferenças nas médias de medidas contínuas foram avaliadas pelo teste *t* de Student, seguido do teste de Mann-Whitney, que − sem exceções − não identificou quaisquer resultados discrepantes (apenas os resultados dos testes paramétricos são relatados). Todas as análises estatísticas foram feitas em um computador pessoal usando o pacote *Statistical Package for the Social Sciences* (SPSS) versão 11.5.1 para Windows.

## RESULTADOS

Cinquenta pacientes do ambulatório de distúrbios adquiridos da fala diagnosticados com AVE e que apresentavam afasia foram selecionados. Foram excluídos sete pacientes, pois dois foram incapazes de completar o protocolo e cinco estiveram ausentes. Os 43 pacientes restantes atenderam aos critérios de inclusão. Assim, 86 orelhas foram examinadas.

### Características gerais

Dezesseis dos 43 pacientes eram mulheres e o grupo tinha uma média de 57,6 anos (desvio padrão − DP=13,2).

### Limiares

Não houve nenhuma diferença estatisticamente significante entre as médias dos limiares das orelhas direita e esquerda para todas as frequências testadas (dados não apresentados).

O audiograma não foi realizado em cinco orelhas: dois indivíduos não conseguiram entender as instruções do teste, e em uma orelha, o meato acústico externo estava obstruído.

A tabela 1 apresenta os limiares de audibilidade para as frequências de 250, 500, 1.000, 2.000, 3.000, 4.000, 6.000 e 8.000Hz.

**Tabela 1:** Média dos limiares de audibilidade para ambos os ouvidos

	250Hz	500Hz	1KHz	2KHz	3KHz	4KHz	6KHz	8KHz
Média	22,8	20,8	17,3	20,8	24,3	33,5	36,9	33,4
DP	11,0	10,8	11,8	16,8	17,2	21,4	22,1	25,5
Mediana	20	20	15	20	20	30	35	30
Mínimo	5	5	5	0	0	5	0	0
Máximo	65	60	65	85	80	100	100	95
n=81								

DP: desvio padrão.

O audiograma foi anormal em 56 ouvidos, indicando algum tipo de perda auditiva. Das 81 orelhas, 25 apresentavam limiares tonais normais em todas as frequências, e 56 apresentavam perda auditiva para pelo menos uma frequência.

Os seguintes tipos de perda auditiva foram observados: 78,6% exibiram perda neurossensorial, 17,8% perda auditiva em apenas uma frequência, e 3,6% perda mista. Em relação aos audiogramas com perda auditiva neurossensorial, observamos uma curva descendente em 50% dos casos, uma curva plana em 19,6%, uma ascendente em 1,8%, e outros tipos em 28,6%.

### Limiares de reconhecimento de fala

Os valores médios do LRF foram 28,6 (DP=12,6) para a orelha direita e 23,1 (DP=7,85) para a orelha esquerda. Figuras tiveram de ser usadas para poder obter o LRF em 12,8% da amostra (11 orelhas).

### Logoaudiometria

O IPRF foi compatível com os resultados do limiar de audiometria tonal liminar em 42,4% das orelhas avaliadas, sendo 15,3% realizado com figuras. No entanto, foi incompatível em 57,6% dos casos porque a possibilidade de responder à fala estava bem pior considerando os limiares auditivos obtidos nesses pacientes em relação à média de limiares auditivos nas frequências de 500, 1.000 e 2.000Hz. Os critérios previamente estabelecidos foram: para uma média entre 0 e 10dB, LRF de 100% seria considerado compatível; entre 11 e 25dB, 96% ou maior; entre 26 e 40dB, 82% ou maior; entre 41 e 55dB, 66% ou maior; entre 56 e 70dB, 56% ou maior; entre 71 e 90dB, 26% ou maior; e para uma média acima de 90dB, 8% ou maior não puderam ser encontrado em 57,6% dos casos.

A logoaudiometria foi realizada usando o método convencional em 63 orelhas (73,3%) e com figuras em 11 orelhas (12,8%). Não houve resposta após a repetição de palavras ou a indicação de figuras em 12 das orelhas testadas (14%).

### Imitância acústica (timpanometria)

Quanto às medidas de imitância acústica, observamos curvas timpanométricas tipo A em 97,6% das orelhas. Curvas do tipo C foram notadas em apenas duas orelhas (2,4%). Curvas timpanométricas do tipo B não foram encontradas.

### Reflexo acústico

Os dados de reflexo acústico foram analisados considerando-se a diferença entre o limiar auditivo e o limiar do reflexo acústico na frequência testada.

A tabela 2 apresenta os resultados de reflexo acústico para ambas as orelhas.

**Tabela 2:** Médias para a diferença entre os limiares dos reflexos auditivos e acústicos em ambos os ouvidos

Reflexo acústico	500Hz	1kHz	2kHz	4kHz
Média	73,68	75,83	74,20	67,92
Mediana	75	75	75	70
Desvio padrão	10,58	10,79	12,91	18,05
Mínimo	50	55	35	30
Máximo	100	105	105	95
Tamanho da amostra	72	70	69	65
Limite inferior	71,24	73,30	71,16	63,54
Limite superior	76,12	78,36	77,25	72,31

A seguir, classificamos a diferença entre o limiar do reflexo e os limiares de audibilidade em quatro categorias e os comparamos para cada frequência. Esses dados estão apresentados na tabela 3.

**Tabela 3:** Distribuição dos valores entre os reflexos e limiares de audibilidade em cada uma as frequências testadas

Reflexo	500Hz n (%)	1kHz n (%)	2kHz n (%)	4kHz n (%)
Menos de 70	21 (24,4)	17 (19,8)	17 (19,8)	31 (36,0)
De 70 a 90	49 (57,0)	49 (57,0)	49 (57,0)	32 (37,2)
Mais de 90	2 (2,3)	5 (5,8)	3 (3,5)	2 (2,3)
Ausente	14 (16,3)	15 (17,4)	17 (19,8)	21 (24,4)

Os valores das EOAT não diferiram entre as orelhas. Os resultados de ambas as orelhas em todos os indivíduos, incluindo o intervalo de confiança, são mostrados na tabela 4.

**Tabela 4:** Valores médios para a amplitude da resposta a emissões otoacústicas evocadas transitórias em ambos os ouvidos

EOAT	1kHz	1,5kHz	2kHz	3kHz	4kHz
Média	7,96	9,40	7,36	7,34	7,16
Mediana	6	9	6	7	7
Desvio padrão	4,67	4,81	4,98	4,68	4,73
Mínimo	1	1	1	1	1
Máximo	22	22	21	16	16
Tamanho da amostra	45	55	45	29	19
Limite inferior	6,59	8,13	5,90	5,64	5,03
Limite superior	9,32	10,67	8,81	9,05	9,28

EOAT: emissões otoacústicas evocadas transitórias.

## DISCUSSÃO

O achado mais significante deste estudo foi a alta taxa de perda auditiva, mais comumente neurossensorial, observada nessa população de pacientes afásicos. A audiometria tonal limiar se mostrou um procedimento extremamente útil, mas as dificuldades de compreensão e/ou de emissão observadas nos pacientes afásicos alteraram a logoaudiometria convencional e apontaram para a necessidade de métodos alternativos de avaliação para tal população.

A população de amostra tinha uma média de idade de 57,48 anos, semelhante a dos relatos de outros estudos,^(11,12)^ embora alguns estudos tenham relatado AVE em pacientes com mais de 60 anos de idade.^(13,14)^

Dois indivíduos foram incapazes de completar os testes convencionais porque não compreendiam as instruções. Esse fato foi justificado pelo fato de pacientes afásicos poderem apresentar distúrbios graves de compreensão.

Em 94,1% dos casos, foi possível avaliar a audição usando audiometria tonal convencional. Assim, pudemos verificar que a audiometria tonal convencional é um método válido de avaliação dessa população e pode ser usada para determinar o grau de perda auditiva periférica que pode agravar os distúrbios de compreensão comuns em pacientes com afasia.

A média dos limiares tonais das frequências altas é maior que a das frequências baixas. A cóclea é uma estrutura helicoidal com aproximadamente 2 giros e 2/3. A base da cóclea tende a vibrar em altas frequências e o ápice tende a vibrar com baixas frequências. Assim, as lesões se iniciam nas frequências mais altas. Além disso, a presbiacusia é causada por deficiências de fluxo sanguíneo para o ouvido interno, causando lesões degenerativas no órgão de Corti. A lesão inicial causa surdez para sons agudos (curva descendente) e perda de discriminação da palavra. A idade da população variou de 22 a 82 anos e, embora a média de idade da população do estudo tenha sido de 57,48 anos, parte da amostra apresentou idade superior a 60 anos de idade. Portanto, esses pacientes poderiam apresentar esse tipo de perda auditiva em decorrência da idade.^(15)^ A presbiacusia é representada por quatro subtipos: sensorial (perda de células ciliadas), neural (perda de neurônios espirais e ganglionares), metabólica (atrofia da estria vascular), e mecânica (espessamento e enrijecimento da membrana basilar).^(16)^ Entretanto, já que múltiplos fatores genéticos e ambientais causam a presbiacusia, a maioria dos casos é de patologia mista e afeta múltiplos tipos celulares.^(17) ^Assim, poderíamos formular a hipótese de que pacientes com problemas vasculares também poderiam ser mais suscetíveis a tais mecanismos.

Além disso, já que o AVE envolve alterações vasculares, é possível que esses adultos com problemas vasculares possam exibir deficiências precoces de fluxo sanguíneo para o ouvido interno.^(18)^ Portanto, nos distúrbios vasculares, pode ocorrer compressão que prejudica determinada área vascularizada, e o indivíduo pode exibir alguns sintomas resultantes da perda de fluxo sanguíneo para essa área.^(19)^

Os resultados de avaliações audiológicas revelaram um audiograma normal em 25 orelhas; 56 orelhas exibiram algum tipo de perda auditiva. Na identificação de casos de perda auditiva, 78,6% foram neurossensoriais, e 17,8% apresentaram uma queda isolada em uma frequência específica (250, 6.000 e/ou 8.000Hz), indicando que alterações do ouvido médio são raras nesses pacientes. Apenas duas orelhas (3,6%) apresentavam perda auditiva do tipo mista.

Na configuração do audiograma, o tipo mais comum foi a curva descendente. Isto pode ter ocorrido porque parte da amostra (47,72%) consistiu de indivíduos com mais de 60 anos de idade, como foi mencionado anteriormente, e esses pacientes podem apresentar presbiacusia, que é normalmente caracterizada por perda neurossensorial de configuração descendente.^(19)^

As medidas de LRF exigem que os pacientes repitam as palavras ouvidas. Sabemos que a população de pacientes afásicos após AVE pode apresentar alteração em sua emissão de fala e frequentemente exibe alterações específicas na tarefa de repetição^(3,20)^ e, com isso alterar as respostas neste tipo de teste. Por esse motivo, as figuras tiveram de ser usadas em 12,8% dos casos. Sabemos que o LRF obtido por meio do álbum de figuras pode ser mais fácil, provavelmente porque se trata de um conjunto fechado (múltipla escolha) com um número limitado de respostas. Assim, é esperado que pacientes afásicos depois do AVE tenham melhores resultados neste tipo de procedimento do que com conjunto “set” aberto^(21)^. Entretanto, mesmo sendo um procedimento mais simples, foi necessário utilizá-lo na população de pacientes afásicos, cujas dificuldades de emissão oral podem dificultar a obtenção de dados do LRF.

Também não foi possível obter o LRF em 14% das orelhas testadas, já que alguns pacientes não entenderam as instruções do teste nem os estímulos em função de sérias dificuldades de compreensão, o que tornou impossível a repetição ou a indicação das figuras representativas das palavras ouvidas.

No IPRF, também foi necessário usar o álbum de figuras em 15,3% dos casos (13 orelhas), mais do que no LRF (12,8%), possivelmente porque palavras monossilábicas com e sem significado foram usadas no IPRF, tornando a tarefa mais difícil. Da mesma forma como foi observado na obtenção do LRF, 14% foram incapazes de repetir ou apontar as figuras das palavras ouvidas. Além disso, os pacientes foram capazes de repetir as palavras em 57,6% dos casos, mas os resultados foram abaixo do esperado para o grau de perda auditiva. Mesmo nos 37,7% dos casos em que o audiograma e o IPRF foram compatíveis, em 15,3% foi utilizado o álbum para obtenção das respostas. Em pacientes afásicos, o comprometimento da compreensão e/ou da emissão é variável. Assim, é possível que o paciente afásico compreenda a tarefa, mas seja incapaz de repetir ou repita com parafasias, o que é considerado um erro no IPRF. O paciente também pode não ser capaz de entender as instruções do teste, ou o paciente pode entender a instrução, mas falhar durante o processamento linguístico de entrada (análise fonológica auditiva,* buffer* fonológico de entrada, léxico fonológico de entrada, e sistema semântico).^(22,23)^ Assim, se considerarmos a complexidade do processamento linguístico, os erros observados durante a repetição poderiam ter múltiplas origens em pacientes afásicos. Embora a tarefa de repetição seja extremamente simples para algumas pessoas sem lesão cerebral, a repetição é extremamente complexa em indivíduos afásicos após AVE. Em suma, a tarefa de repetição pode ser alterada em diversos pontos do processamento linguístico, além da possibilidade de associação com alterações na programação motora da fala, comumente encontrada em pacientes com afasia.^(24,25)^ Em pacientes sem lesão cerebral com comprometimento periférico da audição, há compatibilidade entre o audiograma e IPRF. A incompatibilidade entre os limiares de audibilidade e o IPRF em pacientes afásicos se relaciona com falhas no processamento linguístico, que geram erros na tarefa de repetição. Esses resultados nos fizeram repensar nossos métodos de avaliação da audição em pacientes especiais, a fim de tirar a influência das dificuldades apresentadas por esses pacientes. Nossos resultados também confirmam a importância do uso de tarefas em que o paciente aponte para a resposta, já que pacientes com transtornos graves de compreensão respondem melhor a esse tipo de tarefa.^(26)^

As curvas timpanométricas tipo A foram as mais comumente observadas (97,6%), indicando que essa população raramente tem alteração de orelha média. Parte da população estudada incluiu indivíduos com mais de 60 anos de idade (47,72%), e a perda auditiva neurossensorial acompanhada de curvas timpanométricas tipo A é mais comum neste grupo, já que frequentemente surgem de alterações de orelha interna e/ou nas vias auditivas centrais.^(19)^

Na análise do reflexo acústico, foram considerados os valores referentes a diferença entre o limiar do reflexo acústico e o limiar de audibilidade nas frequências testadas. A média dos valores na frequência mais alta (4.000Hz) foi menor que a média obtida nas outras frequências. Esse resultado pode ser explicado pelo alto número de pessoas com perda auditiva neurossensorial, com uma curva descendente nessa amostra. Sabemos que, na presença de perda auditiva, podemos encontrar o recrutamento. O recrutamento é um sintoma de perda auditiva coclear e diz respeito à sensação psicoacústica rápida e anormal que acompanha o aumento na intensidade do estímulo.

Para todas as frequências testadas, a diferença entre o limiar de reflexo acústico e o limiar de audibilidade ficou, na maioria das vezes, entre 70 e 90dB. Nas frequências de 500, 1.000 e 2.000Hz, houve notável diferença entre essa categoria e as demais, porém, na frequência de 4.000Hz, observamos um número significante da categoria abaixo de 70dB, indicando que o recrutamento era mais comum em 4.000Hz (um forte indicador de lesão coclear), compatível com o fato de que houve maior perda auditiva nas frequências mais altas.

Na análise das emissões otoacústicas, a média de respostas na frequência de 4.000Hz foi menor do que nas demais frequências. Nesses testes, valores de respostas menores foram observados quando o limiar de audibilidade para a frequência estava perto dos limites de normalidade. De fato, a latência das emissões diminui com o aumento da frequência.^(27)^ Já que a maioria dos pacientes exibiu curvas descendentes, os limiares das frequências mais altas foram maiores que os limiares das frequências mais baixas.

A comparação entre a audiometria tonal e as emissões otoacústicas transitórias demonstrou que 19 pacientes tiveram ausência de EOAT em uma ou mais frequências, mesmo com limiares normais de audibilidade. Realmente, estudos anteriores com indivíduos expostos a ruído e com indivíduos submetidos a tratamento quimioterápico com cisplatina^(28-30) ^demonstraram a importância dessa técnica de avaliação para detectar precocemente alteração nas células cocleares, mesmo quando não houve alteração nos limiares de audibilidade avaliados pela audiometria de limiar tonal. Assim, sugerimos que este seja um exame de grande validade, já que detecta precocemente alterações na cóclea, que podem ocorrer como resultado de deficits na irrigação sanguínea. O teste também é importante para propiciar uma ideia da audição dos pacientes quando eles não são capazes de realizar nenhuma das atividades na avaliação audiológica básica.

Neste estudo, observamos perda auditiva em uma ou mais frequências em 56 orelhas, indicando alta prevalência de perda auditiva nessa população. Também observamos que, dessas 56 orelhas, 43 tiveram perda auditiva neurossensorial e 2 exibiram perda auditiva do tipo mista. A configuração audiométrica mais comum foi descendente. Indivíduos com perda auditiva em frequências altas (acima de 2.000Hz) podem exibir dificuldades no reconhecimento da fala.

Dada à importância da audição para o desenvolvimento de aptidões linguísticas e para a eficiência da comunicação, concluímos que a avaliação audiológica nessa população é indispensável, tanto para fins clínicos como para pesquisa, já que é importante diferenciar deficits periféricos de centrais na avaliação e reabilitação de pacientes afásicos após a ocorrência de uma lesão cerebral.

### Limitações do estudo

Este é um estudo transversal, de forma que não é possível identificar, com precisão, a causa da perda auditiva. Neste estudo, apenas pudemos demonstrar que a maioria dos pacientes afásicos apresentou perda auditiva.

## CONCLUSÃO

Houve uma alta taxa de perda auditiva, na maioria das vezes neurossensorial, em pacientes afásicos avaliados neste estudo. As alterações de compreensão e/ou emissão oral, nesses pacientes, alteraram a logoaudiometria convencional e apontaram para a necessidade de usar procedimentos alternativos de avaliação, a fim de fornecer melhor assistência e uma melhor reabilitação de fala/linguagem nessa população.
